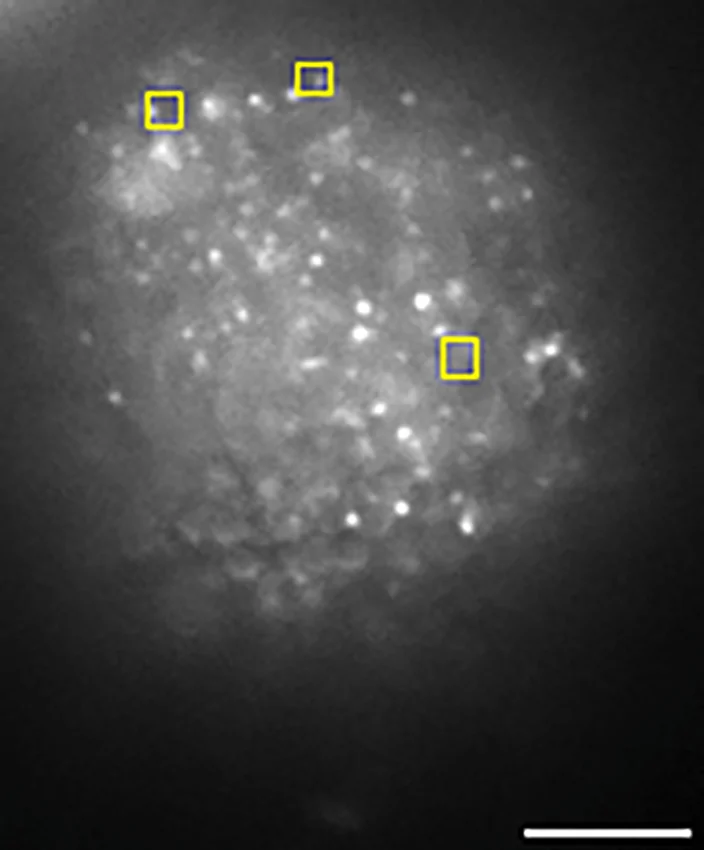# A biosensor platform for scalable and live detection of PYY hormone production from enteroendocrine cells with single-event resolution

**DOI:** 10.1016/j.jbc.2026.113367

**Published:** 2026-07-24

**Authors:** Aanya Hirdaramani, Gary Frost, Aylin C. Hanyaloglu

**Affiliations:** 1 Section of Nutrition, Department Metabolism, Digestion and Reproduction, Imperial College London, London, United Kingdom; 2 Department Metabolism, Digestion and Reproduction, Institute of Reproductive and Developmental Biology, Imperial College London, London, United Kingdom

**Keywords:** obesity, nutrition, G-protein coupled receptors, nutrient-sensing receptors, appetite regulation, intestinal epithelium

## Abstract

Peptide YY (PYY) comprises the secretory repertoire of enteroendocrine L-cells alongside glucagon-like peptide-1 (GLP-1), and positively modulates postprandial satiety, digestion mechanics, and regeneration of the intestinal epithelium. While immortalized GLP-1-secreting human L-cell models support pre-clinical drug discovery, comparable human lines that robustly secrete PYY are lacking, hindering mechanistic studies of its release. We present a biosensor for detection of PYY production and secretion from human enteroendocrine cells *in vitro*. Guided by *in silico* structural prediction, we engineer a superecliptic phluorin (SEP)-tagged PYY, SEP-PYY, that engages native hormone processing machinery and is responsive to canonical nutrient stimuli when expressed in a human enteroendocrine-like cell line, NCI-H716. SEP-PYY production and secretion can be measured by flow cytometry and spectrofluorometric plate readouts, respectively, methods with superior time- and cost-efficacy to current hormone detection methods. Leveraging the pH sensitivity of SEP, we use this biosensor in the detection of single-event hormone exocytosis by total internal reflection microscopy. Finally, we demonstrate the application of our platform in screening ligands of metabolite-sensing Gprotein-coupled receptors that drive SEP-PYY secretion, supporting discovery of druggable and nutrient-driven pathways in metabolic disease.

Peptide YY (PYY) is secreted by enteroendocrine L-cell populations enriched in the distal gut, in response to nutrient and microbiota-derived stimuli (1). Acting through neuropeptide Y (NPY) receptors, PYY regulates central appetite pathways (2), modulates gastrointestinal motility and nutrient absorption (3), and exerts growth factor–like trophic effects on epithelial and endocrine tissues. The anorexigenic effects of PYY are primarily mediated by Y2R (4, 5), and Y2R agonism has demonstrated marked pre-clinical efficacy in reduction of weight loss and energy intake when administered as a co-therapy to GLP-RAs (6, 7), or when targeted *via* multi-receptor agonists (8, 9, 10). However, clinical progression of Y2R agonists has been limited by poor tolerability and high rates of adverse effects (11). Targeting nutrient- and metabolite-sensing GPCRs that drive native PYY release from enteroendocrine L-cells presents an alternative strategy for harnessing the metabolic benefits of PYY whilst potentially avoiding the onset of GI symptoms that arise from systemic activation of central circuits. PYY also promotes colonic epithelial regeneration in *in vitro* models of inflammatory bowel disease (12), further supporting the characterization of agonists that enhance local PYY accumulation in the gut microenvironment.

Although PYY and GLP-1 are both products of the L-cell, these hormones occupy distinct vesicular populations (13), and certain stimuli can selectively promote the release of only one (14, 15). In contrast to the well-characterized mechanisms driving GLP-1 secretion (16, 17, 18, 19, 20, 21), we currently have far scarcer knowledge on druggable targets controlling PYY release from the L-cell. Efforts to screen nutrient and/or pharmacological PYY secretagogues are limited due to a lack of immortalized cell lines that reliably secrete PYY (22). Intestinal organoids-though physiologically representative-require complex differentiation protocols that yield low and variable proportions of enteroendocrine cells (EECs). Moreover, current methods for the detection of gut hormone secretion are both cost- and time-intensive. Here, we describe a novel fluorescent reporter system for measuring PYY from human L-cells, with scalable assay readouts and a functional responsiveness to bioactive nutrient stimuli.

## Results

### *In silico* construct design guides faithful posttranslational processing and targeting of SEP-PYY to secretory granules in a human enteroendocrine cell line

**Figure 1:**
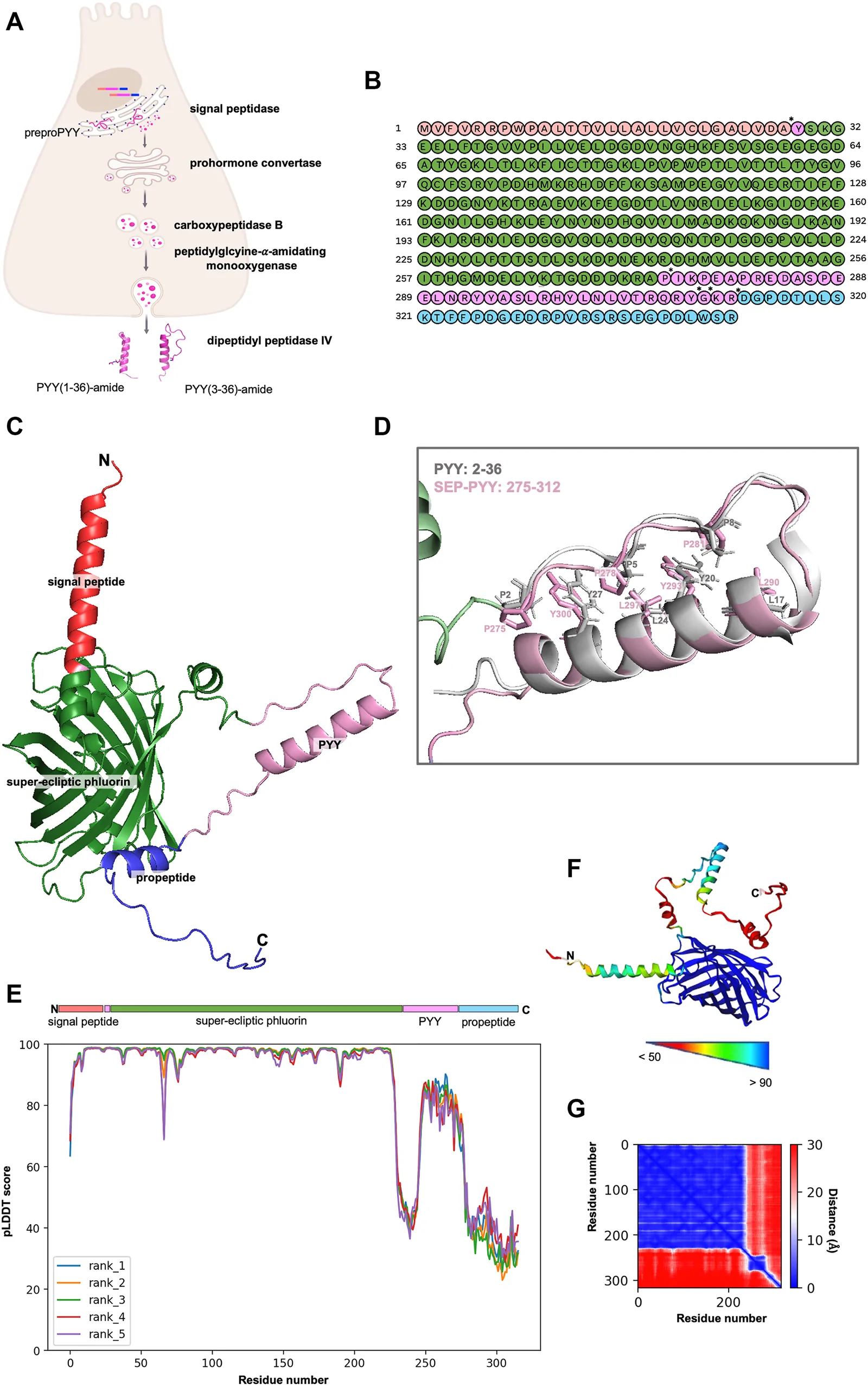
**SEP-PYY construct design preserves native prohormone processing sites and structure of PYY functional domains.***A*, Schematic of post-translational processing of preproPYY in native L-cells, featuring the crystal structure of mature PYY1-36 (PDB: 7RT9). Created by BioRender. Hirdaramani. *A*. (2026). *B*, amino acid sequence of SEP-PYY coloured by domain. *Red*: Signal Peptide, *Pink*: PYY1-36, *Green*: SEP tag, *Blue*: Propeptide. *C*, highest-ranking AlphaFold2 3D structural prediction (Rank 1) of SEP-PYY. *D*, Structural overlay of the PP-fold domain of crystal structure of PYY (PDB: 7RT9) and AlphaFold2 Rank 1 prediction of SEP-PYY, coloured in *grey* and *pink* respectively, with key domain interface residues labelled. *E*, predicted Local Distance Difference Test (pLDDT) Output for SEP-PYY. *F*, SEP-PYY Rank 1 coloured by pLDDT score. *G*, predicted Alignment Error (PAE) output of SEP-PYY Rank 1.

Our aim was to design a PYY biosensor which could faithfully mimic the biosynthesis and secretion of endogenous PYY in human enteroendocrine cells. The pH-sensitive variant of green fluorescent protein (GFP), super-ecliptic phluorin (SEP), was chosen as the fluorescent tag for construct design due to its high signal-to-noise ratio and appropriate pKa for reporting vesicle fusion (23). To ensure tagging of a mature and functional PYY construct, SEP-tagged PYY (SEP-PYY) sequences included the full-length prohormone precursor of PYY (preproPYY). In the rationale design of an insertion point for the SEP tag, we considered three criteria; A) preservation of prohormone enzymatic processing sites required for correct posttranslational processing and targeting of functional PYY to secretory granules (Fig. 1, *A* and *B*) (24) maintenance of the conserved Y2R-binding ‘PP-fold’ domain of PYY (residues 2–8 & 12–33 of PYY1-36) (25, 26) C) integrity of the C-terminal pentapeptide (residues 32–36 of PYY1-36) is crucial for receptor binding potency (27, 28, 29). Based on these criteria, SEP was inserted between Tyrosine1 and Proline2, and *in silico* structural predictions of the candidate sequence were determined by AlphaFold2 (30) (Fig. 1*B*).

Structural prediction of the top scoring model of SEP-PYY (Fig. 1*C*) was superimposed with the crystal structure of human PYY1-36 (Fig. 1*D*) (31), confirming that key inter-residue distances between the N-terminal polyproline core (P2, P5, and P8 of PYY1-36; P275, P278, P281 of SEP-PYY) and the C-terminal helix of the PP-fold (L17, Y20, L24 and Y27 of PYY1-36; L290, Y293, L297, and Y300 of SEP-PYY) were within 0.6 Å of each other (Table S1). Per-residue and domain-level confidence was assessed using predicted local distance difference test (pLDDT) (Fig. 1, *E* and *F*) and predicted alignment error (PAE) scores respectively (Fig. 1*G*). The mean pLDDT score indicated ‘High’ model confidence (averaging 85.6 across models). Residues corresponding to PP-fold (residues 276–312) achieved PAE scores lower than 10 Å, which are consistent with high confidence predictions in the arrangement of this domain. Lower pLDDT and PAE scores were confined to the intrinsically disordered C-terminal tail of SEP (residues 258–275) (32) and the PYY propeptide region (residues 313–342). which lacks homologous structures and exhibits low evolutionary constraints (Fig. S1) (33). Using the SignalP server (34), we obtained a high probability (*p* = 0.8003) prediction of signal peptidase-mediated cleavage of SEP-PYY between positions A28 and Y29, which maps to the respective cleavage site of endogenous preproPYY which frees the mature 1 to 36 form peptide (Fig. S1).

**Figure 2:**
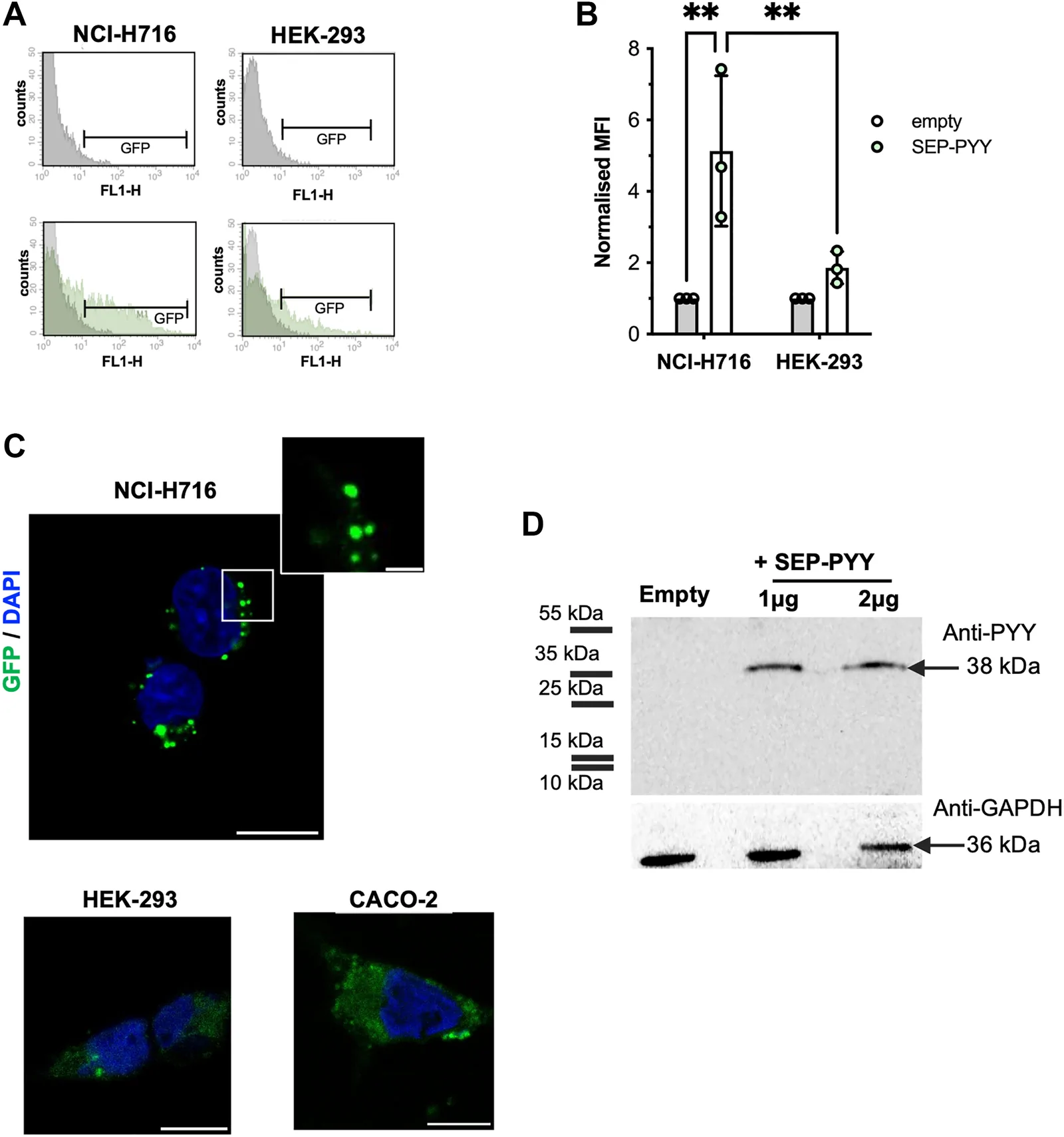
**SEP-PYY is targeted to secretory granules in the human enteroendocrine NCI-H716 cell line.** Protein expression levels of SEP-PYY in transfected NCI-H716 and HEK-293 cell lines were quantified by flow cytometry. *A*, representative histograms showing GFP fluorescence (FL-H) and cell counts in untransfected (*Empty*) and transfected NCI-H716 and HEK-293 samples. *B*, SEP-PYY mean fluorescence intensity (MFI) normalised as fold change over untransfected cells, in NCI-H716 and HEK-293 cells. Symbols represent the mean ± SD of three individual repeats. (∗∗*p* < 0.01, Two-way ANOVA comparing SEP-PYY *versus* empty vector conditions within each cell line and SEP-PYY-transfected NCI-H716 *versus* HEK-293 cells. *C*, confocal images of SEP-PYY expressed in NCI-H716, HEK-293 and Caco-2 cells. Representative images of n = 10 cells, captured across three individual repeats. Scale Bar = 10 μm, Inset Scale Bar = 1 μm. *D*, Western blot of NCI-H716 cell lysates probed with anti-PYY antibody and anti-GAPDH as a housekeeping control. Lysates were prepared from untransfected (*Empty*) cells and cells transfected with 1 μg or 2 μg SEP-PYY plasmid.

We selected the human colonic EEC-like NCI-H716 cell line, which models the nutrient-sensing receptor complement (13, 35) and GLP-1 secretion profiles (36) of human L-cells, to express the SEP-PYY biosensor. Native PYY expression is detectable at the transcript level in this cell line, although at ∼5000 lower abundance relative to proglucagon (13, 15), and several attempts to quantify PYY secretion in this model have either failed to detect secretion (22) or report release following chronic ligand stimulation (13) to pre-load cellular stores (15). SEP-PYY was transiently transfected into NCI-H716 cells, as well a non-enteroendocrine control cell line, HEK-293, and protein expression levels were assayed by flow cytometry (Fig. 2*A*). SEP-PYY protein expression, quantified by Mean Fluorescence Intensity (MFI), was significantly higher in NCI-H716 cells than HEK-293 cells (Fig. 2*B*). Confocal microscopy revealed a distinct vesicular expression profile of SEP-PYY in NCI-H716 cells (Fig. 2*C*), consistent with post-translational processing and targeting into secretory granules. Employing adaptive deconvolution to achieve granule-resolved super resolution. as in our previous work (13), we observed a marked preferential colocalization of SEP-PYY granules with the classic secretory granule marker Chromagranin A (CgA) compared to endosomal marker Early Endosomal Antigen 1 (EEA1) (Fig. S2). This localization further supports a secretory granule identity of SEP-PYY vesicular structures. Transfection of SEP-PYY into two ‘non-EEC’ lines-HEK-293 cells as well as the intestinal absorptive line Caco-2-yielded diffuse cytoplasmic fluorescence (Fig. 2*C*), indicating a dependence of SEP-PYY on endogenous EEC machinery for efficient granule targeting. We next confirmed that this fluorescent signal in NCI-H716 cells was indicative of a full-length tagged SEP-PYY construct, rather than cleaved or non-specific fluorescent derivatives. Western Blotting with an anti-PYY antibody detected a 38 kDa band, corresponding to the molecular weight of SEP-PYY, in lysates from transfected but not untransfected cells (Fig. 2*D*). A band indicative of endogenous PYY(1–36) expression (predicted at 11 kDa), was not observed in any condition, in line with the low native PYY expression in this cell line.

### SEP-PYY production and secretion is responsive to nutrient stimuli

**Figure 3:**
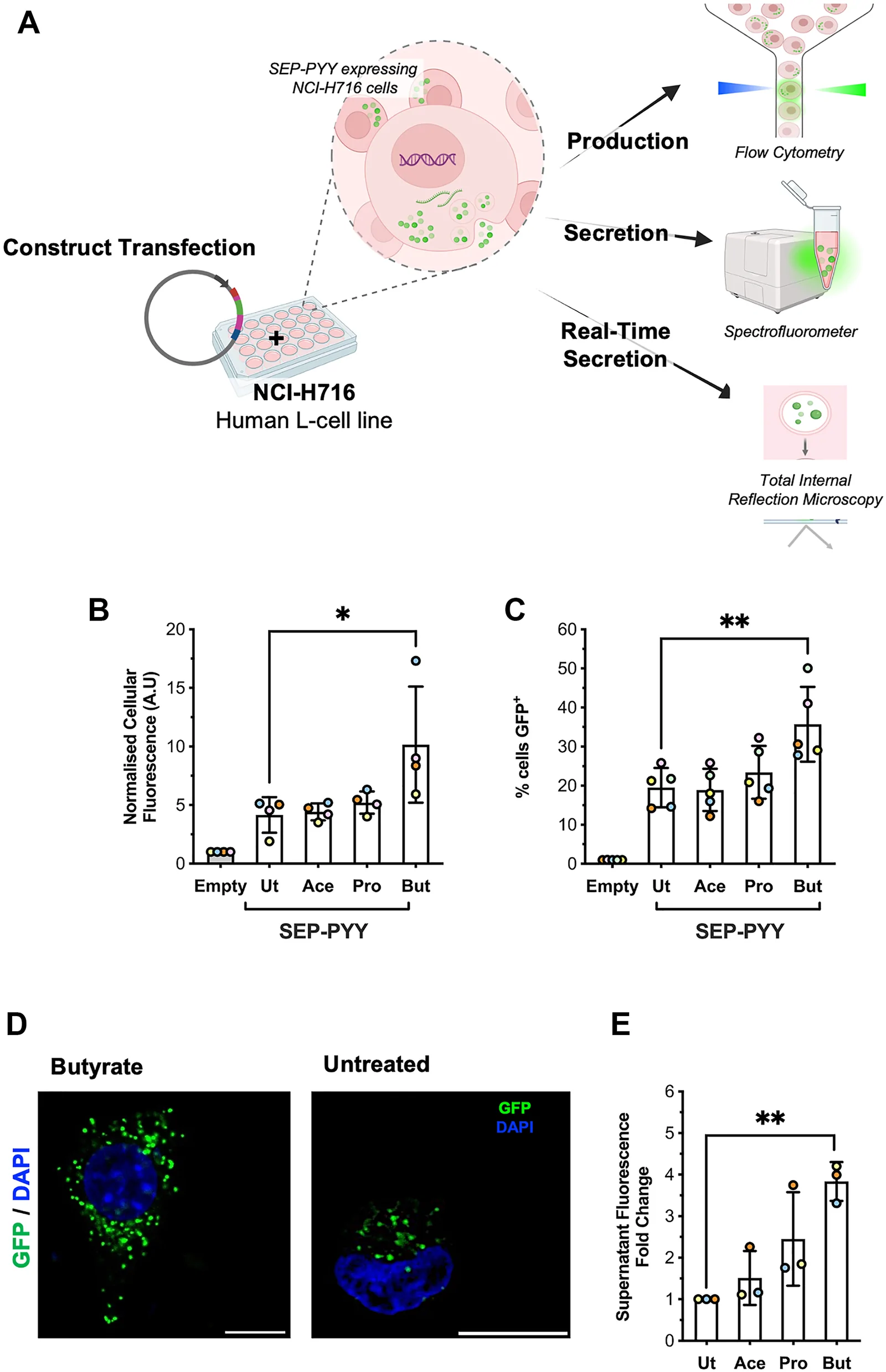
**Butyrate upregulates SEP-PYY production and secretion in NCI-H716 cells.***A*, schematic of workflows for assaying SEP-PYY production and secretion in NCI-H716 cells. Flow cytometric measurements of (*B*) normalized cellular fluorescence and (*C*) % cells GFP-positive in transfected NCI-H716 cells (*Empty*) and NCI-H716 cells transfected for 48 h with SEP-PYY. Transfected cells were untreated (Ut) or treated with 2 mM of Acetate (Ace), Propionate (Pro), or Butyrate (But) for 24 h. Data are presented as fold change over untransfected cells. Symbols represent the mean ± SD of at least n = 4 individual experimental runs. (∗*p* < 0.05; ∗∗*p* < 0.01; One-way ANOVA with Dunnett’s *post hoc* test; SEP-PYY *Ut versus* Ace/Pro/But). *D*, confocal images of SEP-PYY transfected cells with Butyrate treatment, and in Untreated conditions. *E*, supernatant fluorescence of NCI-H716 cells transfected with SEP-PYY for 48 h and treated with 2 mM Ace, Pro or But for 24 h (or *left* Untreated), followed by 2 h incubation in ligand-free secretion buffer. Data are presented as fold change over untransfected cells. Symbols represent the mean ± SD of n = 3 individual experimental runs. (∗∗*p* < 0.01; One-way ANOVA with Dunnett’s *post hoc* test; SEP-PYY Ut *versus* Ace/Pro/But).

We and others have previously demonstrated that the short-chain fatty acid (SCFA) butyrate robustly upregulates PYY in *in vitro* L-cell models (13, 15, 37, 38). To confirm whether SEP-PYY expressing NCI-H716 cells mimic the responsiveness of endogenous PYY to upstream nutrient cues and demonstrate the utility of this biosensor across platforms, we measured SEP-PYY expression by flow cytometry, and secretion by a spectrofluorometer-based plate assay, in response to SCFA stimulation (Fig. 3*A*). We quantified SEP-PYY production by flow cytometric measurements of cellular GFP fluorescence (Fig. 3, *B* and *C*). Butyrate significantly increased both the percentage of cells GFP-positive cells and mean fluorescence intensity (MFI) in SEP-PYY-transfected NCI-H716 cells, consistent with the increased vesicles observed qualitatively *via* confocal microscopy (Fig. 3*D*). In contrast, we did not detect increased SEP-PYY production following treatment with either acetate or propionate, consistent with previous findings that these SCFAs are less efficacious stimulators of native PYY production at a transcriptional/translational level than butyrate (13, 15).

The detection of gut hormone secretion conventionally relies on ELISA or immunoassays, which are cost-and time-intensive, respectively. We leveraged and optimized a spectrofluorometric readout for detection of SEP-PYY secretion from NCI-H716 cells, which outperforms current methods of hormone detection in speed and price. Chronic exposure of each SCFA resulted in a significant increase in SEP-PYY levels secreted into the media following butyrate treatment (Fig. 3*E*).

**Figure 4:**
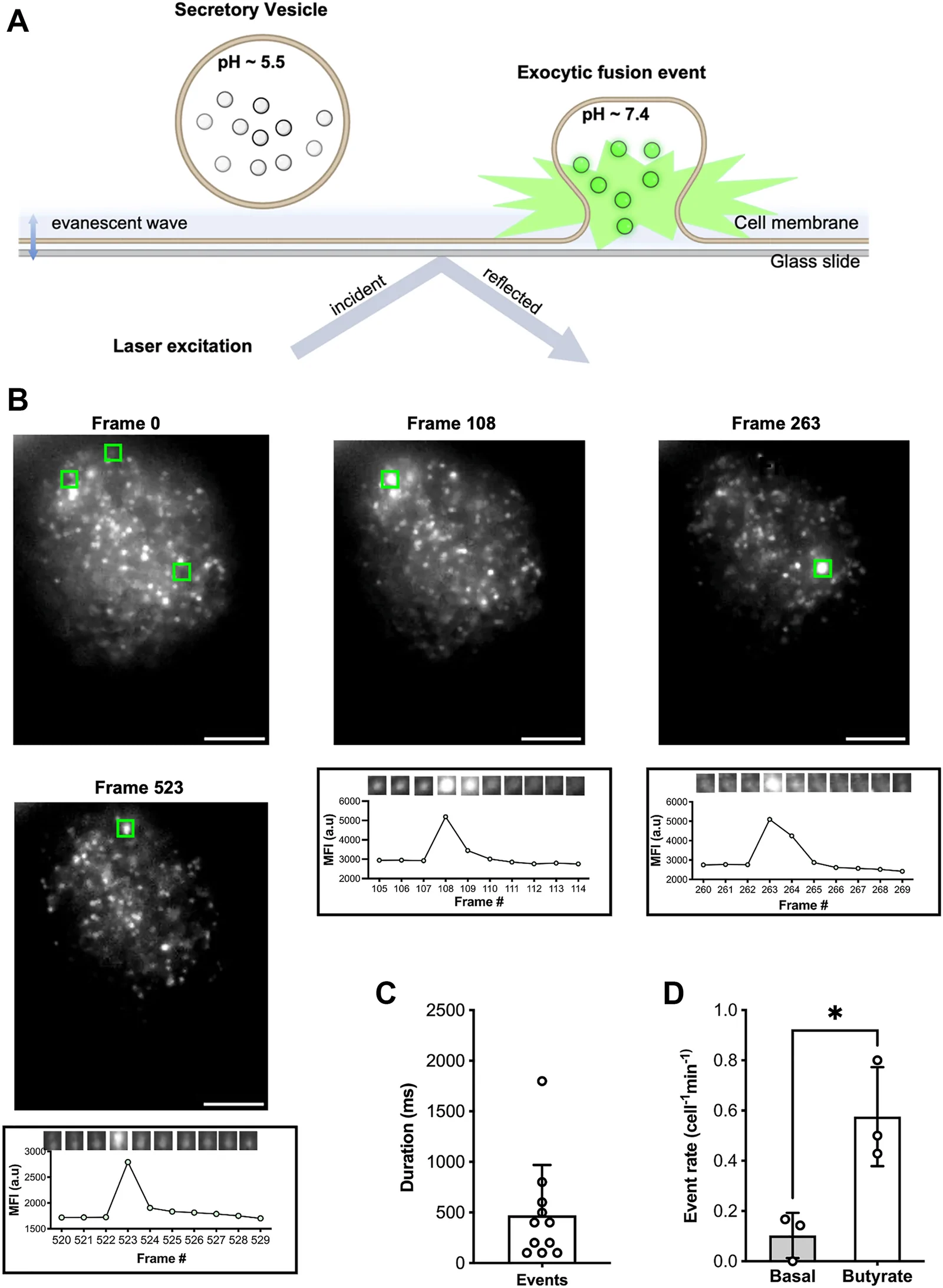
**Visualizing SEP-PYY secretion with single-event resolution using Total Internal Reflection Fluorescence Microscopy (TIRF-M).***A*, schematic of a characteristic single SEP-PYY vesicle exocytic fusion event at the plasma membrane captured by TIRF-M. *B*, representative image sequence of a SEP-PYY-transfected NCI-H716 cell, pre-treated with 2 mM But for 24 h, captured by TIRF-M resolved at 100 ms intervals, for 60 s. *Green* borders indicate regions of interest (ROI) for flash events. Associated traces show the mean fluorescence intensity (MFI) of each flash event sampled at100 ms intervals, alongside zoomed views of each ROI in the indicated frame range. *C*, mean flash event duration in SEP-PYY transfected NCI-H716 cells, captured in n = 11 cells, across n = 3 independent experiments. Error bar represents SD. *D*, quantification of flash events in SEP-PYY transfected cells pre-treated with butyrate, or without treatment (Basal). n = 22 to 24 cells were imaged per condition, across n = 3 independent experiments. Symbols represent the mean ± SD of each repeat. (∗∗*p* < 0.01, *t* test; Ut *versus* But).

The use of total internal reflection microscopy (TIRF-M) in capturing fusion of exocytic vesicles with the plasma membrane has been valuable in investigating phasic release profiles of other endocrine peptides (39, 40), and phase-specific defects in pathophysiological conditions (40). The pKA of SEP confers a key advantage in reporting exocytotic events; plasma membrane fusion of acidic secretory vesicles (∼pH 5.5) with the extracellular environment (∼pH 7.4) coincides with a marked increase in phluorin fluorescence (Fig. 3*A*). We captured single-event SEP-PYY exocytosis in SEP-PYY-transfected NCI-H716 cells using TIRF-M, acquiring videos at 100 ms intervals over 60 s (Supplementary Video 1). Flash events appeared as prominent increases in fluorescence, exceeding a threshold defined as the mean background intensity + 2 standard deviations, consistent with established criteria for analysis of exocytosis events by fluorescence techniques (41) (Fig. 4*B*). The mean duration of individual events was 472.2 ms (Fig. 4*C*), consistent with previous TIRFM studies of peptide release (39, 40). Using this approach, we quantified a ∼2.5-fold increase in flash event frequency with butyrate treatment, consistent with the effect of butyrate on enhanced secreted SEP-PYY fluorescence (Fig. 4*D*). TIRF-M studies of insulin (40) and GLP-1 (39) secretion have shown that flash events can arise from either pre-docked granule pools already resident at the plasma membrane or from ‘newcomer’ granules that are trafficked from deeper in the cell towards immediately before membrane fusion. In our recordings, flash events arose exclusively from newcomer granules, which appeared *de novo* at the plasma membrane before fusion.

### SEP-PYY can be used to screen nutrient and pharmacological agonists of L-cell GPCRs for their ability to act as acute secretagogues

**Figure 5:**
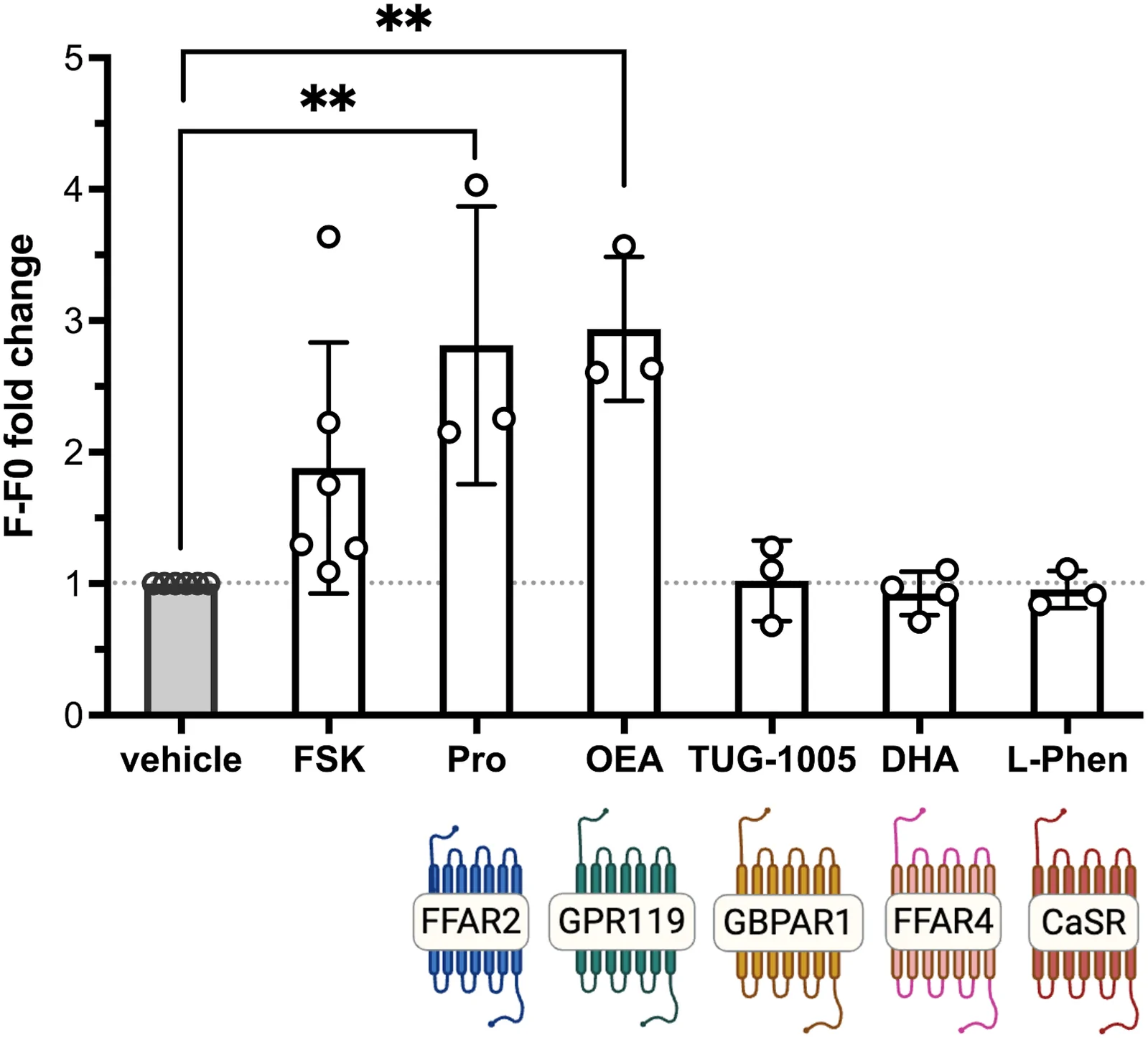
**SEP-PYY secretion is differentially regulated by agonists of nutrient-sensing GPCRs in NCI-H716 cells.** Supernatant fluorescence of NCI-H716 cells transfected with SEP-PYY for 48 h and treated for 2 h with 10 μM Forskolin (FSK), 10 mM Propionate (Pro), 10 μm oleoylethanolamide (OEA), 10 μm TUG-1005, 100 μM docasohexanoic acid (DHA) and 30 mM L-phenylalanine (L-Phen) in ligand-free secretion buffer. Data are presented as background-corrected fold change over vehicle. Symbols represent the mean + SEM of at least n = 3 individual experimental runs. ∗∗*p* < 0.01; one-way ANOVA with Dunnett’s *post hoc* correction.

A diverse complement of nutrient-sensing GPCRs drive enterohormone release in native L-cells, presenting multiple targetable pathways for synergistic stimulation of secretion (42). We tested the responsiveness of the SEP-PYY biosensor to acute stimulation with ligands of GPCRs which are endogenously expressed in L-cells, and have previously been implicated in driving GLP-1 release from NCI-H716 cells (13, 21, 43, 44, 45) (Fig. 5) (17, 46, 47, 48, 49). SEP-PYY-transfected cells were treated for 2 h with stimuli of either free fatty acid receptor 2 (FFAR2), free fatty acid receptor 4 (FFAR4), bile acid receptor (GBPAR1), GPR119 or calcium-sensing receptor (CaSR). We detected a significant increase in fluorescence with either the FFAR2 agonist propionate or the GPR119 lipid agonist oleoylethanolamide (OEA) (Figs. 5 and S3), indicating that although these receptors are all known to induce GLP-1 secretion, they may differ in their ability to induce secretion of PYY.

## Discussion

Here, we establish a scalable *in vitro* system for investigating real-time, stimulus-evoked PYY profiles in human EECs using a SEP-tagged PYY biosensor. We show that the reporter is dependent on native enteroendocrine machinery for processing and targeting into secretory granules, and stimulated by canonical nutrient stimuli, enabling inference of physiologically relevant hormone output. Our optimized workflows provide an approximate fivefold reduction in assay time (∼20 min readout) and ninefold reduction in per-sample cost (∼1USD) relative to ELISA-based detection, while maintaining sensitivity for both biosynthesis and secretion. This system could also be readily adapted to more complex epithelial models such as intestinal organoids and re-engineered to interrogate alternative hormone cargos.

EECs express a wide repertoire of nutrient-sensing GPCRs that represent attractive opportunities for selectively modulating gut hormone release (1). Previous work (13, 50), including our own, shows that GLP-1 and PYY exist in distinct vesicle pools in enteroendocrine L-cells, and exhibit non-identical stimulus-secretion coupling. We therefore screened SEP-PYY expressing cells with a panel of GPCR agonists previously implicated in driving GLP-1 release from L-cells (13, 21, 43, 44, 45), we found that only acute activation of FFAR2 and GPR119 elicited robust PYY release, revealing a degree of input selectivity *via* mechanisms that are likely beyond heterotrimeric G protein coupling profile alone, but could be exploited to fine-tune enterohormone profiles while limiting off-target central effects. Moreover, leveraging the rich signaling diversity of the GPCR superfamily—including subcellular compartmentalization (13, 51, 52), receptor cross-talk (53, 54), and ligand-biased signalling (55)—offers superior routes to precision control of gut hormone secretion.

We demonstrate the use of this platform paired with TIRF-M in reporting live single-event hormone exocytosis. Moreover, our live-cell imaging uncovered distinct granule dynamics for PYY compared to GLP-1. Previous imaging studies have reported a biphasic exocytosis profile of glucose-stimulated GLP-1 secretion, which originates from pre-docked granule pools, followed by “newcomers” (39). In our TIRF-M recordings, pre-docked SEP-PYY granule pools are visible, but both basal and ligand-evoked flash events arose exclusively from newcomers. This further supports the concept that GLP-1 and PYY secretion are regulated in distinct manners from separate secretory pools but raises the possibility that ligand-specific patterns of PYY granule recruitment and fusion exist. Future studies using this platform could define a broader repertoire of ligand-specific PYY granule profiles, and test whether selective mobilization of particular granule pools can further enhance overall hormone output. The pH-sensitivity of SEP enabled selective detection of granule fusion events at the plasma membrane by TIRF, while also permitting multi-platform analyses by high-throughput readouts for biosynthesis and secretion. Although sample pH was tightly controlled across assays, future work might engineer a larger suite of complementary biosensors with photostability and robust fluorescence for improved signal-to-noise and dynamic range for flow cytometry and microplate reader assay formats.

We have validated that SEP-PYY production and secretion remain responsive to butyrate, a well-established secretagogue of endogenous PYY (13, 15, 37, 38). As SEP-PYY is expressed from a constitutive promoter, our findings suggest a translational and/or post-translational action of butyrate on PYY output, rather than direct transcriptional control of the transgene promoter. A mechanistic investigation of butyrate-mediated regulation of PYY was beyond the scope of this study, but prior work implicates activation of the GPCR Free Fatty Acid Receptor 2 (FFAR2) and HDAC inhibition in driving butyrate’s effects on PYY expression and L-cell differentiation (15, 37, 48). Our platform is compatible with pharmacological and genetic perturbations of candidate pathways (for example G-protein signaling, MAPK cascades and epigenetic regulators) enabling future studies to dissect how upstream butyrate, or other candidate stimuli, are integrated at the level of PYY biosynthesis, processing, and/or granule exocytosis. Moreover, coupling this biosensor with live advanced imaging modalities will facilitate temporally resolved analysis of mechanisms governing PYY production, granule trafficking and exocytosis, and may help identify potential phase-specific defects in conditions associated with lowered PYY release (56, 57).

Forskolin was included as a benchmark stimulus for measuring SEP-PYY secretion by microplate reader-based methods, as per previous reports that agents which induce cAMP elevation drive gut hormone secretion (58). Moreover, previous studies in primary human colonic tissue and isolated L-cells have shown that forskolin and propionate typically increase PYY secretion by approximately 2-fold over basal (59, 60), which is consistent with the dynamic range observed in our assay. However, we observed a variation in forskolin responses which might be concentration-related; our assays employed 10 uM compared to previous reports utilizing concentrations up to 100 uM (61) or in combination with IBMX (58) to stimulate GLP-1 secretion. Future studies might use higher forskolin concentrations to engage stronger cAMP stimulation. Importantly, ligands such as propionate and OEA, which directly engage endogenous GPCRs, produced responses that were similar or exceeded this forskolin condition, highlighting that GPCR ligands may be preferable positive controls for mechanistic interrogation of SEP-PYY signaling in future studies. The efficacy of OEA in driving PYY secretion from human L-cells has not yet been directly examined; however, activation of its cognate receptor GPR119 has been implicated as a significant positive modulator of gut hormone secretion, including PYY, *via* small-molecule agonists such as K-833 (49). Comparative data on the relative potencies of different PYY secretagogues in human L-cells remain limited, partly owing to the lack of PYY-secreting human cell lines. Our results therefore primarily serve to illustrate that the assay can resolve ligand- and pathway-specific differences in PYY output across a dynamic range comparable to that reported for native hormone secretion.

Although we have demonstrated that SEP-PYY replicates enteroendocrine-restricted faithful expression and largely recapitulates the functional behaviors of endogenous PYY, we acknowledge that a biosensor requires overexpression of an exogeneous construct, and although a range of ligands were assessed in this study, it may distort native secretory dynamics, such as with vesicle targeting and release, thereby skewing quantitative measures of basal and stimulus-evoked output. Future studies could employ gene-editing approaches to label endogenous PYY, although this approach in turn has its own limitations in terms of off-target effects.

Despite the heightened popularity of gut hormone receptor analogues in obesity, diabetes and beyond, these agents have important limitations. Gastrointestinal disturbances are common, and their benefits require long-term usage because weight gain is frequent following discontinuation (62). In contrast, bariatric surgery achieves durable weight loss, which has been associated with postoperative elevation of both PYY & GLP-1 secretion in these patients (63), implicating enhanced endogenous gut hormone secretion in mediating favorable metabolic outcomes. In order to mimic the sustained benefits of bariatric surgery, next-generation therapeutics could converge on identifying ligands to promote enterohormone secretion from native EECs, rather than relying on exogeneous analogues. Creation of tools, such as the PYY biosensor in this study, could aid such efforts in addition to providing further understanding of the triggers, mechanisms and extracellular environmental requirements that drive PYY secretion in the gut, both physiologically and in disease.

## Experimental procedures

### Cell culture

NCI-H716 cells (ATCC, CCL-251) were maintained in suspension in RPMI-1640 (Sigma-Aldrich) containing 2 g/L D-glucose and 2 mM glutamine supplemented with 10% (v/v) fetal bovine serum (Merck) and 100 U/ml penicillin/streptomycin (Sigma-Aldrich) (complete RPMI). Two days before experiments, cells were seeded onto culture plates that were pre-coated with Cultrex Basement Membrane Extract (170 μl/cm^2^) (R&D Systems) diluted to 0.5 mg/ml. HEK-293 (ATCC, CRL-1573) and Caco-2 (ATCC, CRL-2102) cells were grown as an adherent monolayer in T-75 flasks in DMEM containing 4.5 g/L D-glucose and 4 mM glutamine supplemented with 10% (v/v) fetal bovine serum (FBS) and 100 U/ml penicillin/streptomycin (+/+ DMEM).

### Construct design and molecular cloning

Custom gene fragments containing the human PYY sequence (GenBank; NG_023338.2) tagged with super-ecliptic pHluorin (Addgene; pMOS005) were synthesized *de novo* by Twist Bioscience. Amino acid sequences of SEP-PYY constructs were input to AlphaFold2 (v2.) for structural predictions. Five model predictions were generated, with 3 recycles for structural refinement. Model confidence was assessed using pLDDT scores and PAE matrices. Structural preservation of functional domains between endogenous human PYY and the highest confidence (rank 1) SEP-PYY models was evaluated by measurement of inter-residue distances on Pymol (v). Signal peptidase I cleavage sites were predicted using SignalP5.0 software. Gene fragments were subcloned into pcDNA 3.1+/+ mammalian expression vector using *XbaI* and *AfeI* restriction enzyme sites.

### Transfections

SEP-PYY constructs were expressed in NCI-H716 cells by transient reverse transfection using Lipofectamine 2000 reagent. SEP-PYY-Lipofectamine 2000 mixes were prepared (1 μg DNA:5 μl Lipofectamine/well) and transferred to 6-well plates pre-coated with Matrigel (0.5 mg/ml), and cell suspensions were then added immediately. For transfection of SEP-PYY into the HEK-293 cell line, cells were plated and grown to 75 to 85% confluency prior to transfection. SEP-PYY-Lipofectamine 2000 mixes were prepared as above and added dropwise to cell culture media. Media was changed 16 to 20 h after transfections, and fresh media contained respective SCFAs in the case of chronic SCFA treatments. Cells were grown for an additional 24 h, till 70% to 80% confluent. Reagent volumes and cell number were scaled down based on the surface area of culture plates used for specific assay formats.

### Flow cytometry

SEP-PYY production was assessed by flow cytometry. Cells were washed twice with cold phosphate-buffered saline (PBS) with Ca^2+^ (PBS-Ca^2+^) and harvested in FACs buffer (2% FBS in PBS). Samples were centrifuged for 5 min at 1118*g* at 4 °C, resuspended in FACs buffer and transferred to polypropylene round bottom FACs tubes. Data were acquired using a FACS Calibur Flow cytometer. The parameters of the gating region of interest were determined using readings with untransfected cells.

### Fluorometer-based secretion assay

SEP-PYY secretion was assessed by measurement of fluorescence with a microplate reader-based protocol. On the day of the assay, cells were washed once with PBS and incubated with respective ligands diluted in Krebs buffer (supplemented with 0.2% BSA) for 2 h at 37C. Secretion buffer was then collected, immediately buffered with 15 mM HEPES (pH 7.4) and spun down for 10 min at 800*g* at room temperature. The solution was then transferred to 96-well clear-bottom black-walled microplates (Corning), and fluorescence was measured on a LUMIstar OPTIMA microplate reader with optical filters for excitation and emission set to 485 nm and 520 nm, respectively, using bottom optics. Wells were scanned using orbital averaging of four measurements per well, and detector gain was set to 70% to avoid saturation. Raw fluorescent values were background-subtracted (using no-cell treatment controls) and normalized to signals obtained from untransfected cells in parallel.

### Confocal microscopy

For confocal microscopy, glass coverslips (13 mm) were added to the bottom of wells prior to Matrigel coating, reverse transfections and cell seeding. On the day of the assay, cells were washed with cold PBS-Ca^2+^ prior to fixation with 4% paraformaldehyde for 20 min at room temperature. For staining intracellular markers, cells were then permeabilized with 0.2% Triton-X in PBS-Ca^2+^ for 20 min at room temperature prior to blocking for 1 h at room temperature in 2% FBS in PBS-Ca^2+^ (imaging blocking buffer). Primary antibodies for Chromogranin A (rabbit multiclonal, Abcam, ab283265) or Early Endosomal Marker 1 (rabbit monoclonal, Invitrogen, MA5-14794) were diluted in blocking buffer at 1:50 and 1:1000 dilutions, respectively, and cells were stained overnight at 4 °C. Next, cells were washed thrice with PBS-Ca^2+^ and incubated with anti-rabbit secondary antibody Alexa Fluor conjugate (Invitrogen, A-21245) diluted in blocking buffer at room temperature for one hour. Cells were washed thrice again, and coverslips were mounted using Fluoromount G onto clear glass slides. Slides were imaged on a Stellaris 8 Inverted confocal microscope (Leica) using the LasX software. For colocalization analysis, adaptive deconvolution by Leica LIGHTNING software was employed during acquisition to achieve super-resolution, and Mander’s Overlap Coefficient of SEP-PYY granules with intracellular markers were computed using the JaCoP plugin in Fiji.

### TIRF microscopy

Total internal reflection fluorescence microscopy was conducted on a NanoImager S from ONI (Oxford Nanoimaging; ONI) with cells plated on ibidi μ-Slide 8 well glass bottom chambers. 30 min before imaging, cell media was changed to Krebs buffer supplemented with 0.2% BSA, containing respective ligands. Frames were captured on a closed-loop piezo stage with a 100x NA 1.4 oil immersion objective, and SCmos camera with excitation 405 nm, 488 nm, 561 nm and 640 nm lasers, and the temperature at the Nanoimager was controlled at 37 °C. Samples were brought into TIRF mode by adjustment of the angle of incidence of laser excitation. Frames were captured every 100 ms (10 frames per second) for 1 min per cell and 488 nm laser power at or below 30% during imaging, and at or below 10% during setup to avoid photobleaching. Time-lapse videos were analyzed in ImageJ (FIJI). For classification of flash events, a per-cell detection threshold was set as 2 standard deviations above the pre-event baseline mean fluorescence and only transients exceeding this threshold were segmented into ROIs.

### Western Blotting

Transfected NCI-H716 cells were harvested and lysed with RIPA lysis buffer. Cell lysates were mixed with 4× Laemmli Sample buffer with 5% 2-mercaptoethanol and heated at 100 °C for 10 min. Samples were resolved by SDS-PAGE at 150 V for 2 h, and proteins were transferred to 0.22 μm nitrocellulose membranes at 100 V for 90 min at 4 °C. Membranes were blocked in 5% non-fat dry milk in TBS-Tween (Western blocking buffer). After washing, membranes were incubated with an anti-PYY antibody (rabbit polyclonal, bioRT, orb579781) at a 1:250 dilution in blocking buffer, overnight at 4 °C. Membranes were washed and then incubated with HRP-conjugated anti-rabbit secondary antibody (Invitrogen, 31460) at 1:10,000 dilution in blocking buffer for 1 h at room temperature. Membranes were developed with Immobilon Forte Western HRP Substrate and imaged using ImageQuant software. Band density was quantified and normalized to GAPDH.

### Statistical analysis

All statistical analyses were carried out as indicated in figure legends on GraphPad Prism. Unpaired *t*-tests were used to compare two groups. One-way ANOVA with Dunnett’s *post hoc* test was used for comparisons of multiple groups to the untreated control. Two-way ANOVA was used for experiments involving multiple groups and conditions.

## Materials availability

SEP-PYY construct is available from the lead contact under a materials transfer agreement with Imperial College London.

## Data availability

The data in this study shall be made available upon request from the corresponding author.

## Supporting information

This article contains supporting information.

## Conflict of interest

The authors declare that they have no conflicts of interest with the contents of this article.

## Figures and Tables

**Supplementary Video:**