# Supplementary material for: A biosensor platform for scalable and live detection of PYY hormone production from enteroendocrine cells with single-event resolution
Source: J Biol Chem. 2026 Jul 24;302(9):113367. doi: 10.1016/j.jbc.2026.113367 (PMC13507795; doi:10.1016/j.jbc.2026.113367)
Supplement: Supplementary file 1 [file mmc1.pdf]

Supporting Information

Table S1

**PYY1-36**		**SEP-PYY**	
**Residue pair**	**Cα-Cα (Å)**	**Residue pair**	**Cα-Cα (Å)**
P2-Y27	5.9	P275-Y300	5.9
P5-Y27	7.7	P278-Y300	7.5
P5-L24	5.7	P278-L297	5.8
P5-Y20	6.0	P278-Y293	6.6
P8-Y20	6.6	P281-Y293	7.1
P8-L17	6.3	P281-L290	6.4

Measured distances in angstroms between key structure-function residue pairs of NMR-resolved structure of PYY (PDB: 2DEZ) and AlphaFold2 3D prediction of SEP-PYY visualised on Pymol.

Figure S1

a.

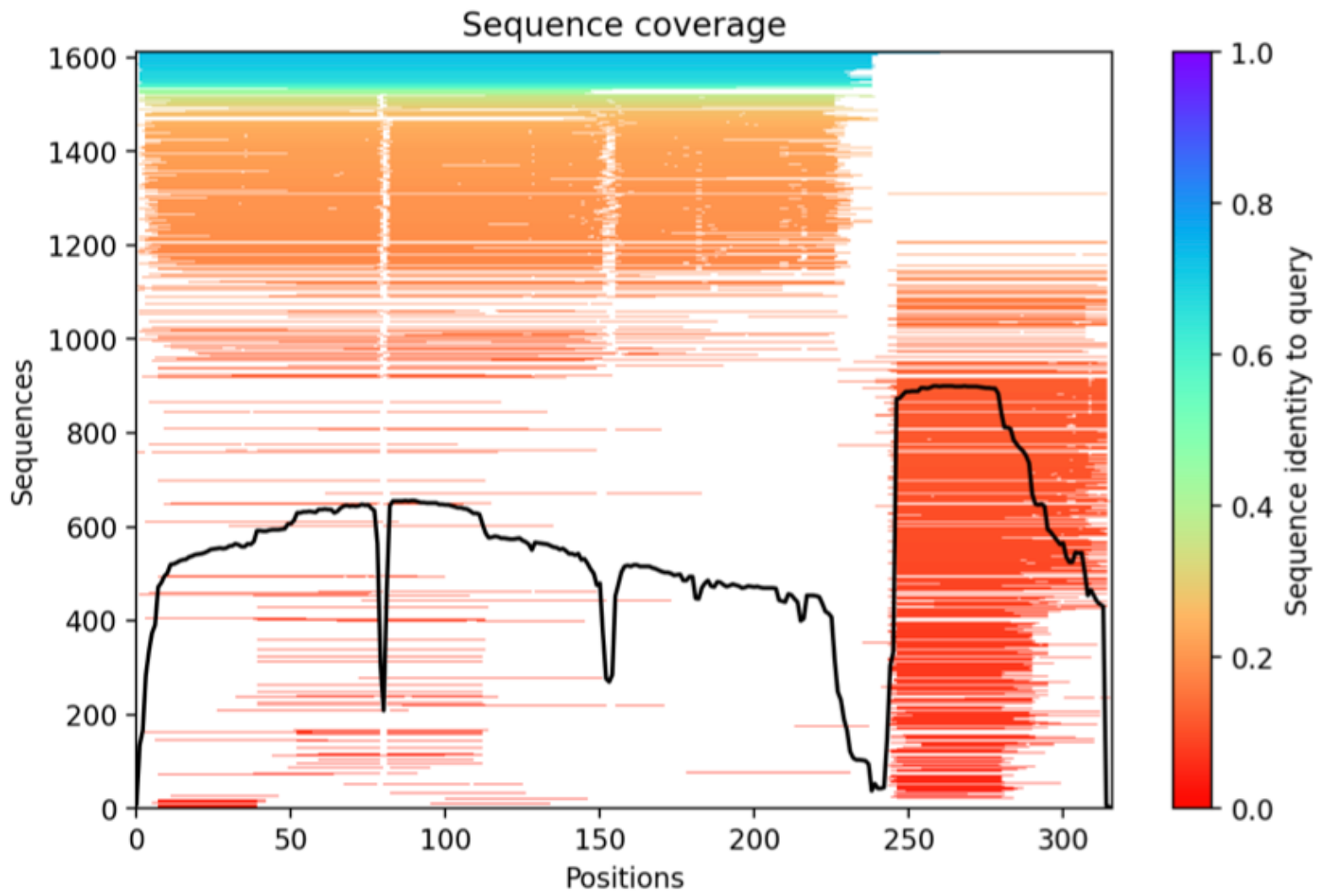

b.

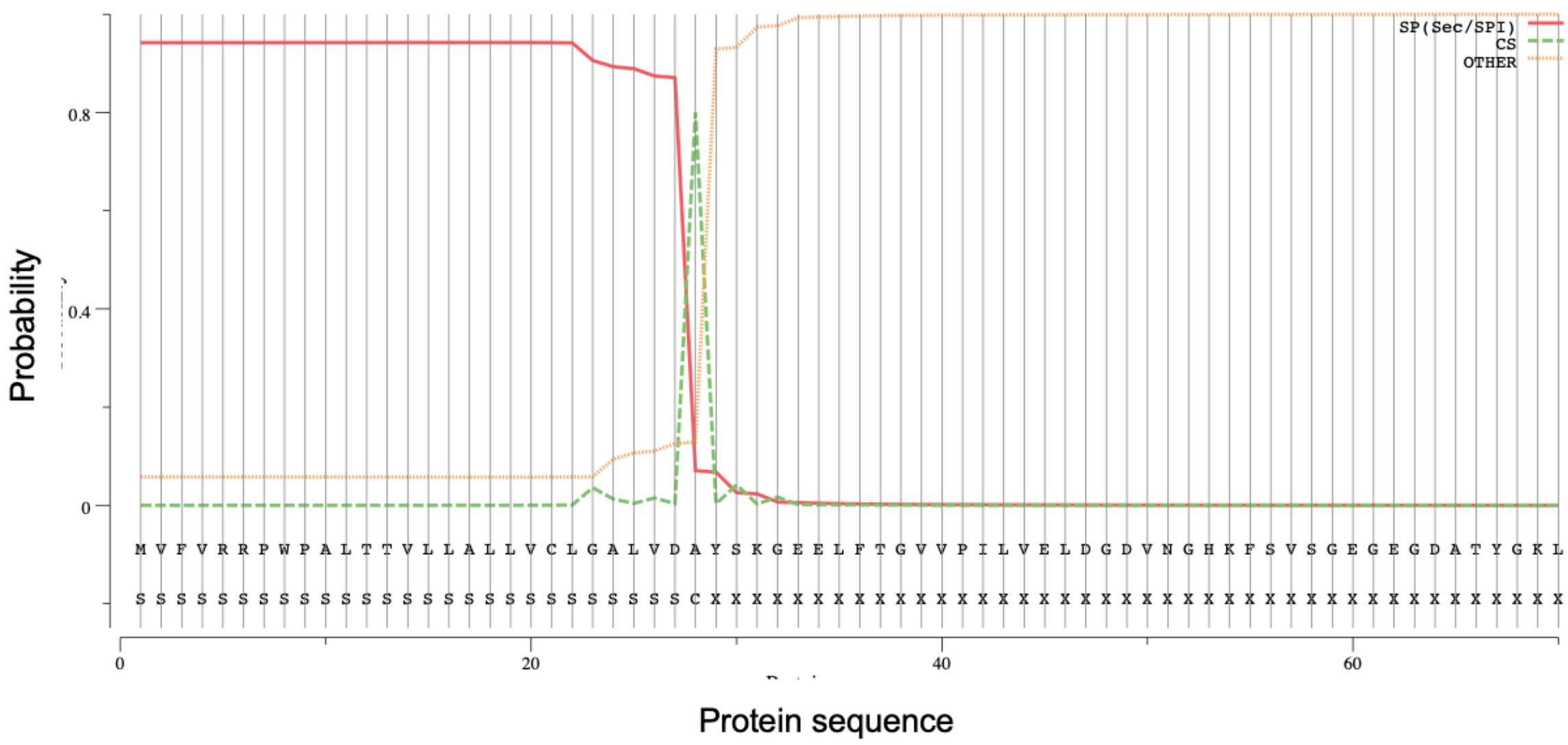

a) AlphaFold2 MSA sequence coverage plot of SEP-PYY.
b) SignalP 5.0 predicted probability of signal peptide sequence (red solid line), cleavage site (dashed green line) or other (dotted orange line) along residues 0-70 of SEP-PYY.

Figure S2

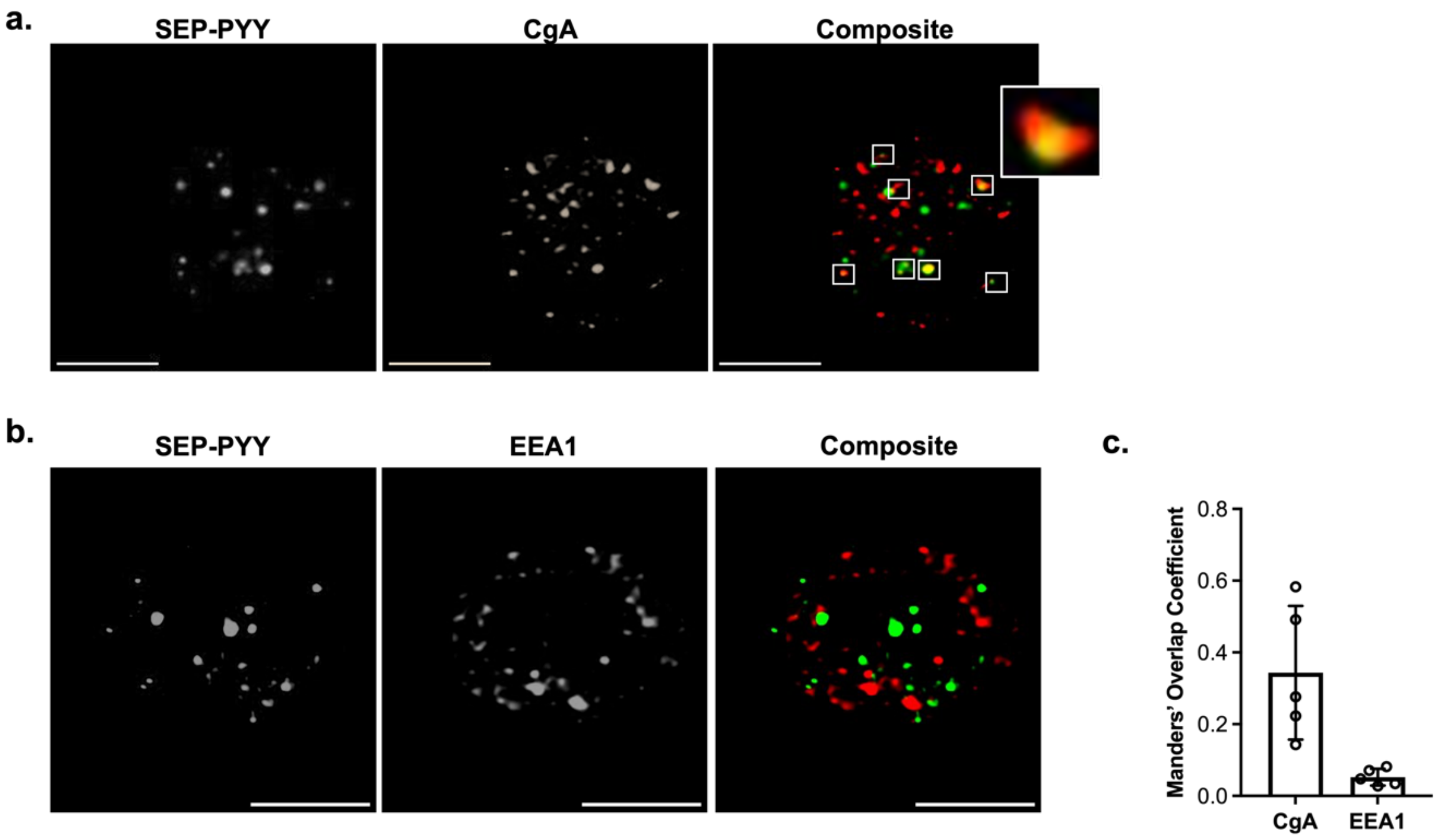

Confocal images of SEP-PYY-transfected NCI-H716 cells stained for a) Chromagranin A (CgA) or b) Early Endosomal Antigen 1 (EEA1). n=5 cells were captured per condition. Scale bar = 10uM. C) Mander's Overlap coefficient of SEP-PYY with CgA or EEA1. Symbols represent values for individual cells, bars represent mean ± SD.

Figure S3

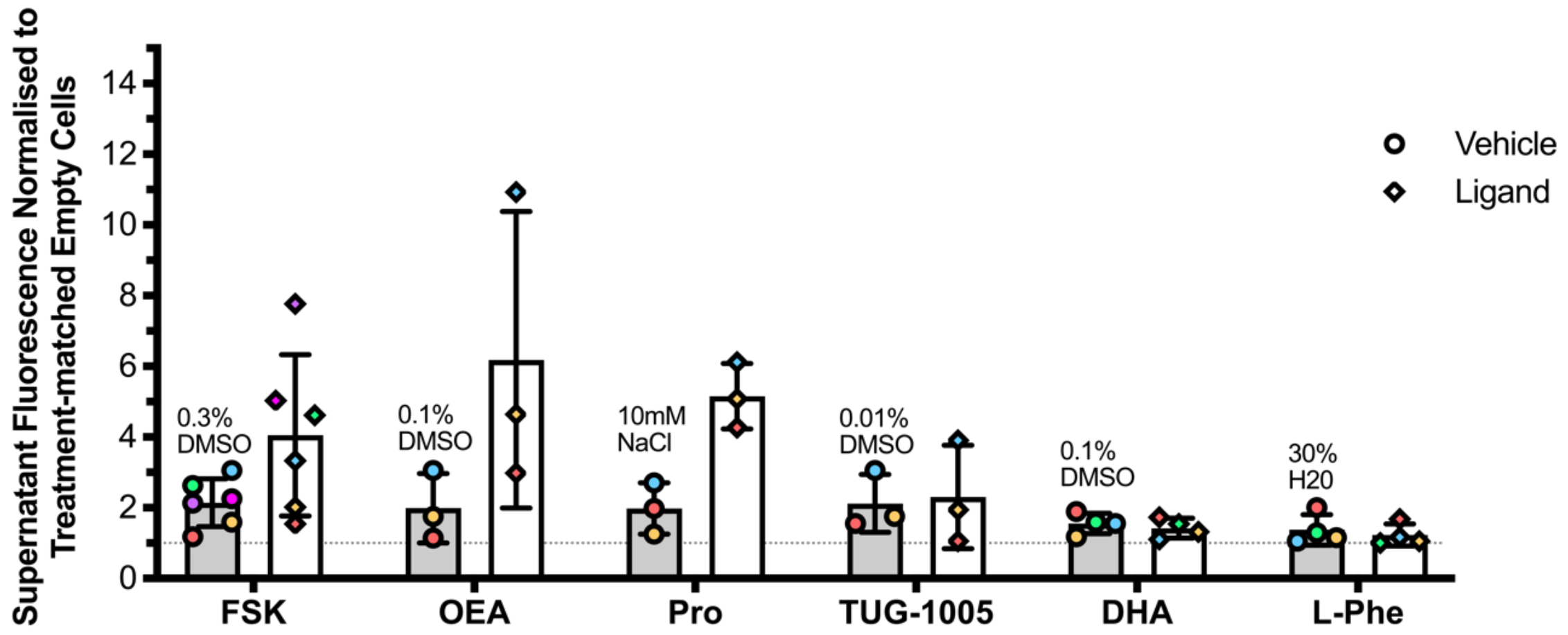

Supernatant fluorescence of SEP-PYY-transfected NCI-H716 cells treated for 2 hours with ligand; 10uM Forskolin (FSK), 10mM Propionate (Pro), 10uM oleoylethanolamide (OE), 10uM TUG-1005, 100uM docasohexanoic acid (DHA) and 30mM L-phenylalanine (L-Phen) or respective vehicle. Data are presented as background-corrected fold change over untransfected cells. Symbols represent the mean ± SD of individual biological repeats, run in at least technical triplicates. Symbol colours represent corresponding biological repeats within vehicle-ligand pairs.

Video S1

Live video of SEP-PYY transfected NCI-H716 cell captured by Total Internal Reflection Fluorescence Microscopy (TIRF-M), pre-treated with butyrate (2mM, 24h). Cells were imaged live at 100ms intervals (10fps) for 5 minutes. ROIs of flash events are indicated by yellow inset borders.
